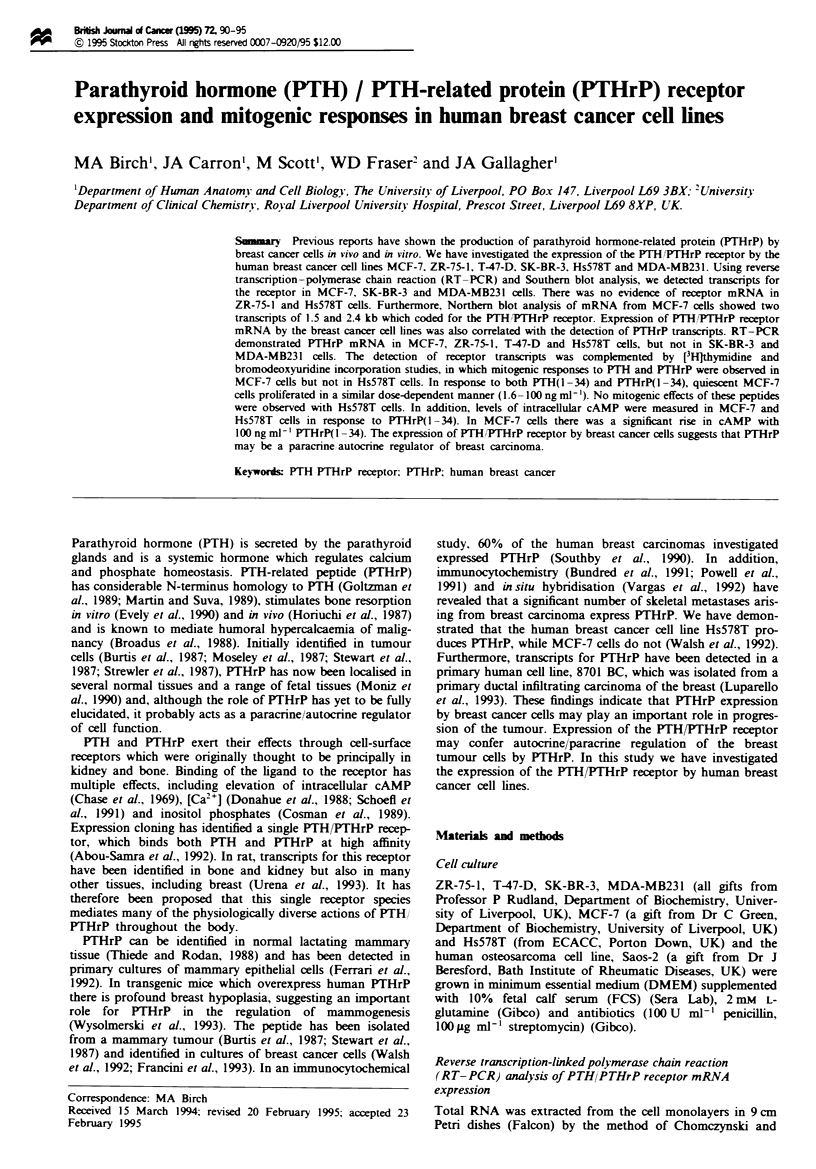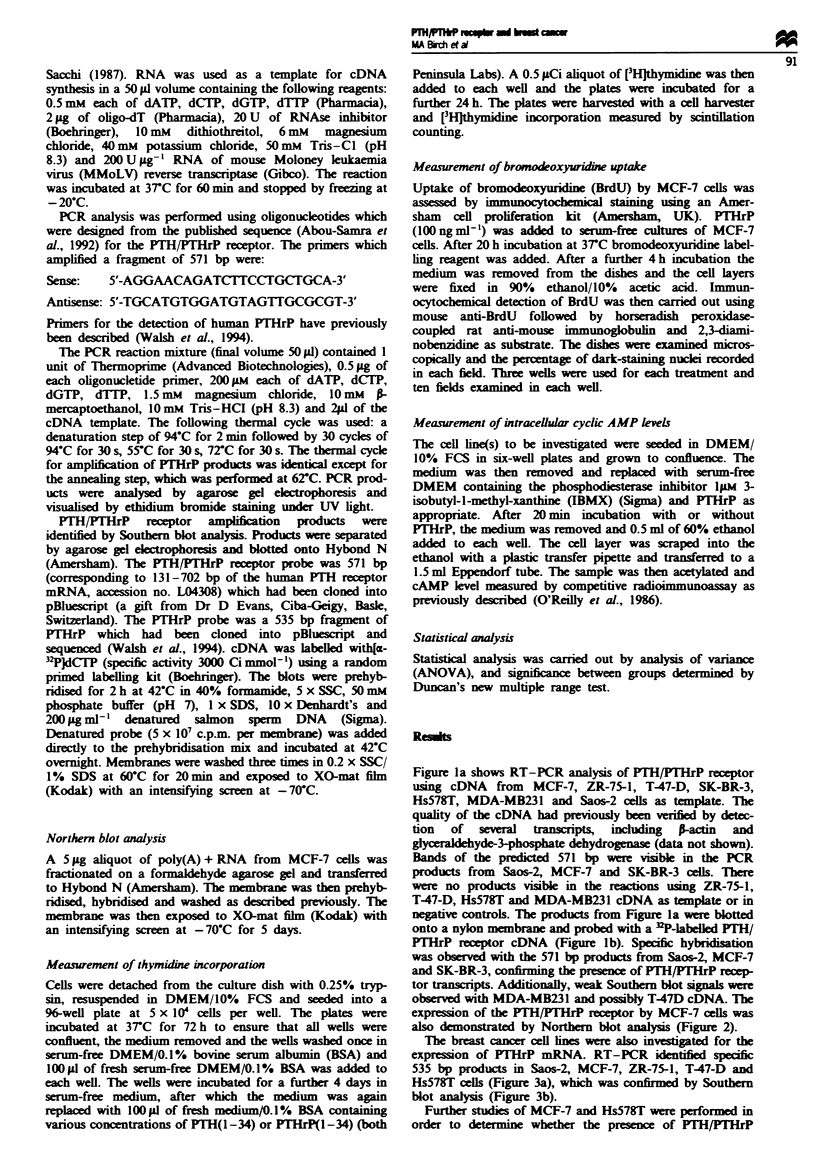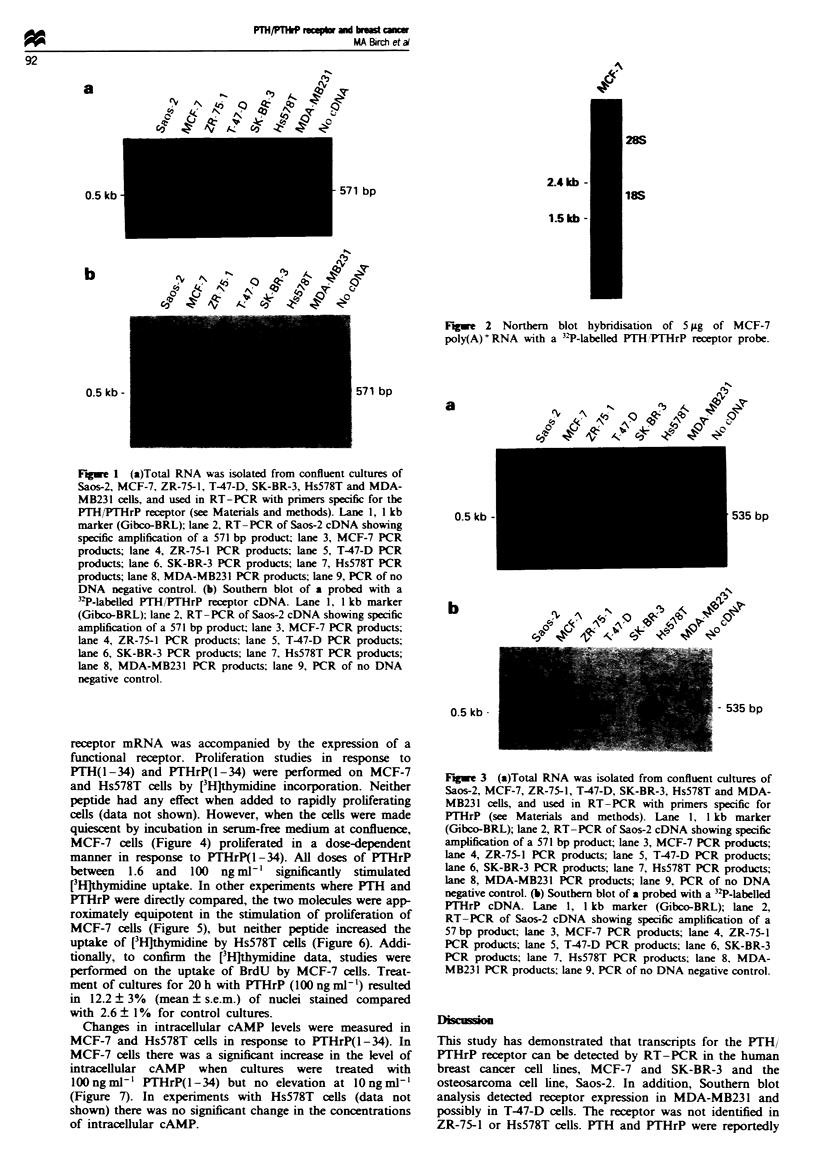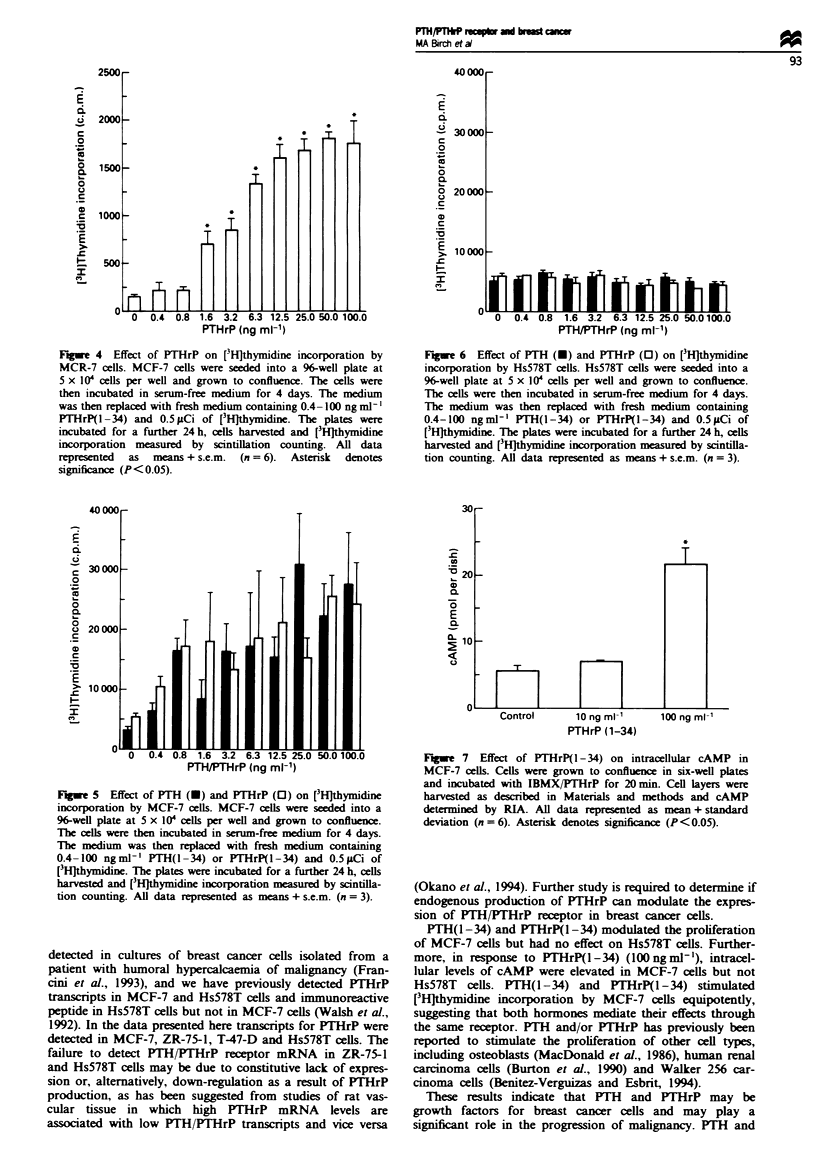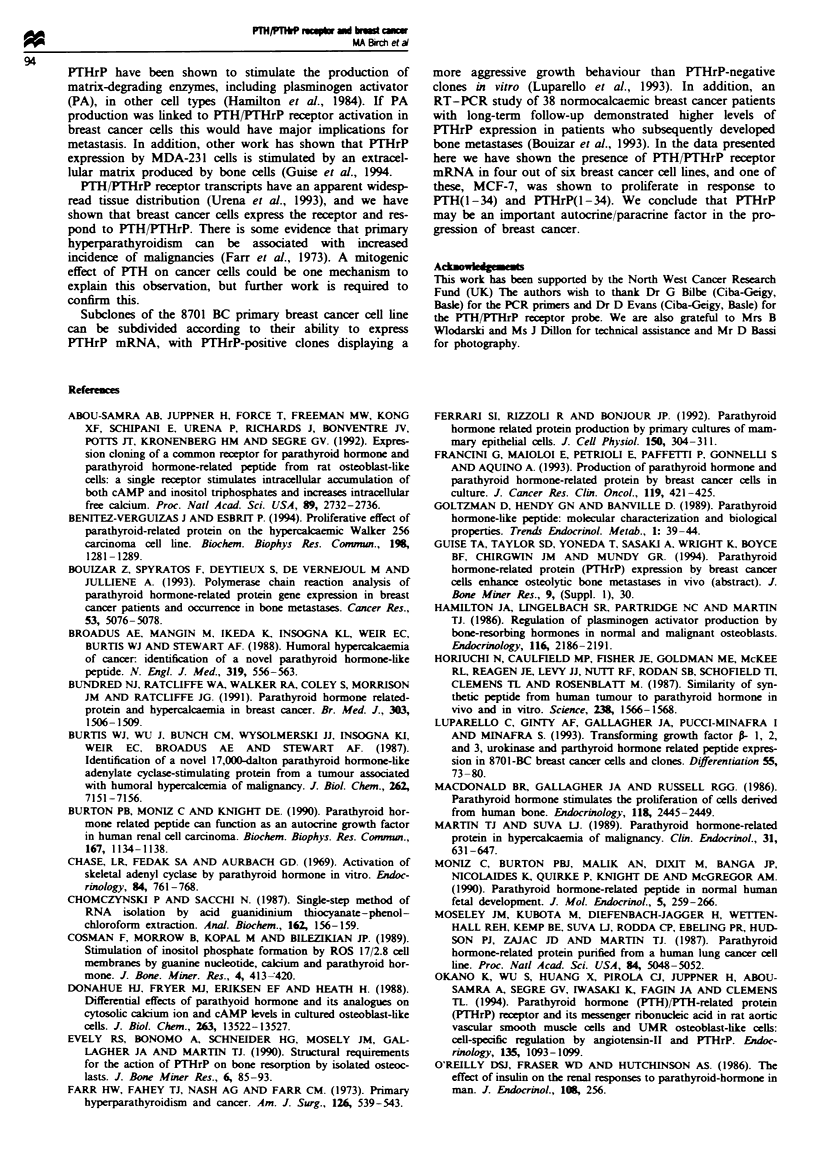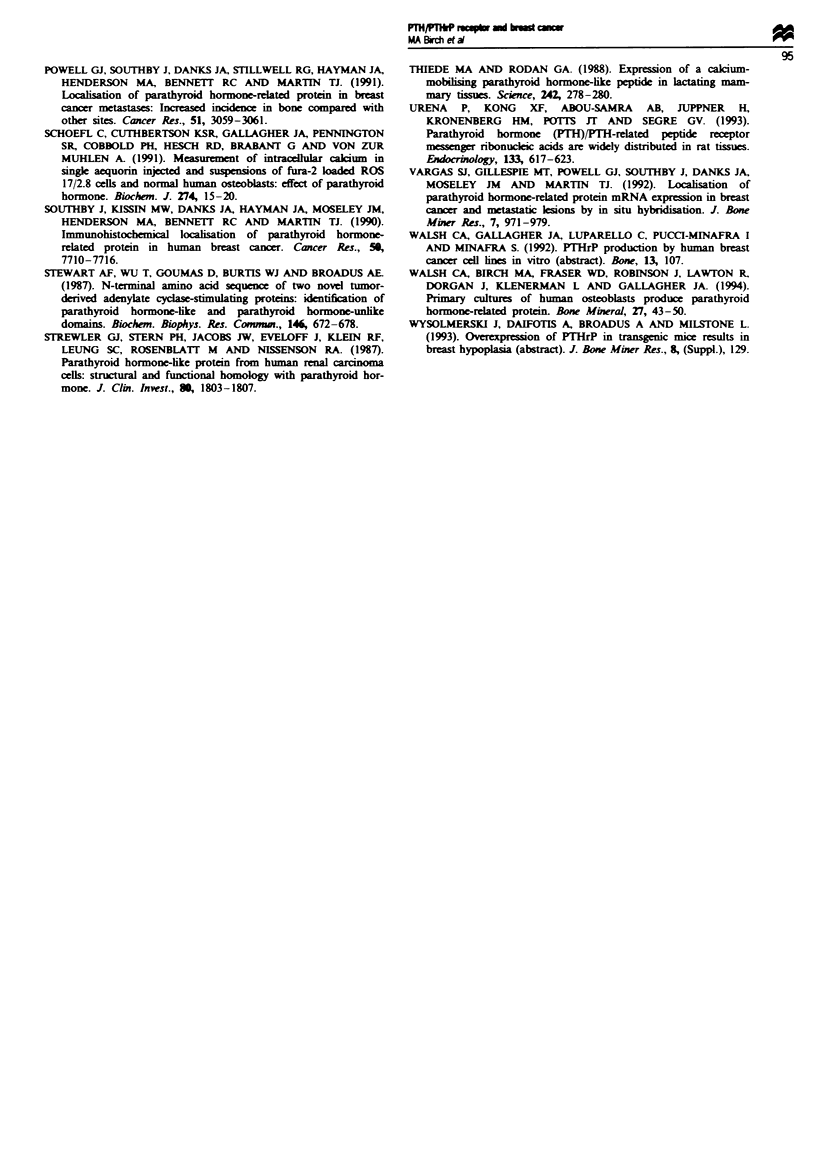# Parathyroid hormone (PTH)/PTH-related protein (PTHrP) receptor expression and mitogenic responses in human breast cancer cell lines.

**DOI:** 10.1038/bjc.1995.282

**Published:** 1995-07

**Authors:** M. A. Birch, J. A. Carron, M. Scott, W. D. Fraser, J. A. Gallagher

**Affiliations:** Department of Human Anatomy and Cell Biology, University of Liverpool, UK.

## Abstract

**Images:**


British Journal of Cancer (1995) 72, 90-95

© 1995 Stockton Press All rights reserved 0007-0920/95 $12.00

### Parathyroid hormone (PTH) / PTH-related protein (PTHrP) receptor expression and mitogenic responses in human breast cancer cell lines

MA Birch[1], JA Carron[1], M Scott[1], WD Fraser[2] and JA Gallagher[1]

[1]*Department of Human Anatomy and Cell Biology, The University of Liverpool, PO Box 147, Liverpool L69 3BX;* [2]*University Department of Clinical Chemistry, Royal Liverpool University Hospital, Prescot Street, Liverpool L69 8XP, UK.*

**Summary** Previous reports have shown the production of parathyroid hormone-related protein (PTHrP) by breast cancer cells *in vivo* and *in vitro*. We have investigated the expression of the PTH/PTHrP receptor by the human breast cancer cell lines MCF-7, ZR-75-1, T-47-D, SK-BR-3, Hs578T and MDA-MB231. Using reverse transcription-polymerase chain reaction (RT-PCR) and Southern blot analysis, we detected transcripts for the receptor in MCF-7, SK-BR-3 and MDA-MB231 cells. There was no evidence of receptor mRNA in ZR-75-1 and Hs578T cells. Furthermore, Northern blot analysis of mRNA from MCF-7 cells showed two transcripts of 1.5 and 2.4 kb which coded for the PTH/PTHrP receptor. Expression of PTH/PTHrP receptor mRNA by the breast cancer cell lines was also correlated with the detection of PTHrP transcripts. RT-PCR demonstrated PTHrP mRNA in MCF-7, ZR-75-1, T-47-D and Hs578T cells, but not in SK-BR-3 and MDA-MB231 cells. The detection of receptor transcripts was complemented by [$^3$H]thymidine and bromodeoxyuridine incorporation studies, in which mitogenic responses to PTH and PTHrP were observed in MCF-7 cells but not in Hs578T cells. In response to both PTH(1-34) and PTHrP(1-34), quiescent MCF-7 cells proliferated in a similar dose-dependent manner (1.6-100 ng ml$^{-1}$). No mitogenic effects of these peptides were observed with Hs578T cells. In addition, levels of intracellular cAMP were measured in MCF-7 and Hs578T cells in response to PTHrP(1-34). In MCF-7 cells there was a significant rise in cAMP with 100 ng ml$^{-1}$ PTHrP(1-34). The expression of PTH/PTHrP receptor by breast cancer cells suggests that PTHrP may be a paracrine/autocrine regulator of breast carcinoma.

**Keywords:** PTH/PTHrP receptor; PTHrP; human breast cancer

Parathyroid hormone (PTH) is secreted by the parathyroid glands and is a systemic hormone which regulates calcium and phosphate homeostasis. PTH-related peptide (PTHrP) has considerable N-terminus homology to PTH (Goltzman *et al.*, 1989; Martin and Suva, 1989), stimulates bone resorption *in vitro* (Evely *et al.*, 1990) and *in vivo* (Horiuchi *et al.*, 1987) and is known to mediate humoral hypercalcaemia of malignancy (Broadus *et al.*, 1988). Initially identified in tumour cells (Burtis *et al.*, 1987; Moseley *et al.*, 1987; Stewart *et al.*, 1987; Strewler *et al.*, 1987), PTHrP has now been localised in several normal tissues and a range of fetal tissues (Moniz *et al.*, 1990) and, although the role of PTHrP has yet to be fully elucidated, it probably acts as a paracrine/autocrine regulator of cell function.

PTH and PTHrP exert their effects through cell-surface receptors which were originally thought to be principally in kidney and bone. Binding of the ligand to the receptor has multiple effects, including elevation of intracellular cAMP (Chase *et al.*, 1969), [$Ca^{2+}$] (Donahue *et al.*, 1988; Schoefl *et al.*, 1991) and inositol phosphates (Cosman *et al.*, 1989). Expression cloning has identified a single PTH/PTHrP receptor, which binds both PTH and PTHrP at high affinity (Abou-Samra *et al.*, 1992). In rat, transcripts for this receptor have been identified in bone and kidney but also in many other tissues, including breast (Urena *et al.*, 1993). It has therefore been proposed that this single receptor species mediates many of the physiologically diverse actions of PTH/PTHrP throughout the body.

PTHrP can be identified in normal lactating mammary tissue (Thiede and Rodan, 1988) and has been detected in primary cultures of mammary epithelial cells (Ferrari *et al.*, 1992). In transgenic mice which overexpress human PTHrP there is profound breast hypoplasia, suggesting an important role for PTHrP in the regulation of mammogenesis (Wysolmerski *et al.*, 1993). The peptide has been isolated from a mammary tumour (Burtis *et al.*, 1987; Stewart *et al.*, 1987) and identified in cultures of breast cancer cells (Walsh *et al.*, 1992; Francini *et al.*, 1993). In an immunocytochemical study, 60% of the human breast carcinomas investigated expressed PTHrP (Southby *et al.*, 1990). In addition, immunocytochemistry (Bundred *et al.*, 1991; Powell *et al.*, 1991) and *in situ* hybridisation (Vargas *et al.*, 1992) have revealed that a significant number of skeletal metastases arising from breast carcinoma express PTHrP. We have demonstrated that the human breast cancer cell line Hs578T produces PTHrP, while MCF-7 cells do not (Walsh *et al.*, 1992). Furthermore, transcripts for PTHrP have been detected in a primary human cell line, 8701 BC, which was isolated from a primary ductal infiltrating carcinoma of the breast (Luparello *et al.*, 1993). These findings indicate that PTHrP expression by breast cancer cells may play an important role in progression of the tumour. Expression of the PTH/PTHrP receptor may confer autocrine/paracrine regulation of the breast tumour cells by PTHrP. In this study we have investigated the expression of the PTH/PTHrP receptor by human breast cancer cell lines.

### Materials and methods

### Cell culture

ZR-75-1, T-47-D, SK-BR-3, MDA-MB231 (all gifts from Professor P Rudland, Department of Biochemistry, University of Liverpool, UK), MCF-7 (a gift from Dr C Green, Department of Biochemistry, University of Liverpool, UK) and Hs578T (from ECACC, Porton Down, UK) and the human osteosarcoma cell line, Saos-2 (a gift from Dr J Beresford, Bath Institute of Rheumatic Diseases, UK) were grown in minimum essential medium (DMEM) supplemented with 10% fetal calf serum (FCS) (Sera Lab), 2 mM L-glutamine (Gibco) and antibiotics (100 U ml$^{-1}$ penicillin, 100 μg ml$^{-1}$ streptomycin) (Gibco).

### Reverse transcription-linked polymerase chain reaction (RT-PCR) analysis of PTH/PTHrP receptor mRNA expression

Total RNA was extracted from the cell monolayers in 9 cm Petri dishes (Falcon) by the method of Chomczynski and

Correspondence: MA Birch
Received 15 March 1994; revised 20 February 1995; accepted 23 February 1995

Sacchi (1987). RNA was used as a template for cDNA synthesis in a 50 μl volume containing the following reagents: 0.5 mM each of dATP, dCTP, dGTP, dTTP (Pharmacia), 2 μg of oligo-dT (Pharmacia), 20 U of RNAse inhibitor (Boehringer), 10 mM dithiothreitol, 6 mM magnesium chloride, 40 mM potassium chloride, 50 mM Tris-Cl (pH 8.3) and 200 U $\mu g^{-1}$ RNA of mouse Moloney leukaemia virus (MMoLV) reverse transcriptase (Gibco). The reaction was incubated at 37°C for 60 min and stopped by freezing at −20°C.

PCR analysis was performed using oligonucleotides which were designed from the published sequence (Abou-Samra *et al.*, 1992) for the PTH/PTHrP receptor. The primers which amplified a fragment of 571 bp were:

Sense: 5′-AGGAACAGATCTTCCTGCTGCA-3′

Antisense: 5′-TGCATGTGGATGTAGTTGCGCGT-3′

Primers for the detection of human PTHrP have previously been described (Walsh *et al.*, 1994).

The PCR reaction mixture (final volume 50 μl) contained 1 unit of Thermoprime (Advanced Biotechnologies), 0.5 μg of each oligonucletide primer, 200 μM each of dATP, dCTP, dGTP, dTTP, 1.5 mM magnesium chloride, 10 mM β-mercaptoethanol, 10 mM Tris-HCI (pH 8.3) and 2μl of the cDNA template. The following thermal cycle was used: a denaturation step of 94°C for 2 min followed by 30 cycles of 94°C for 30 s, 55°C for 30 s, 72°C for 30 s. The thermal cycle for amplification of PTHrP products was identical except for the annealing step, which was performed at 62°C. PCR products were analysed by agarose gel electrophoresis and visualised by ethidium bromide staining under UV light.

PTH/PTHrP receptor amplification products were identified by Southern blot analysis. Products were separated by agarose gel electrophoresis and blotted onto Hybond N (Amersham). The PTH/PTHrP receptor probe was 571 bp (corresponding to 131-702 bp of the human PTH receptor mRNA, accession no. L04308) which had been cloned into pBluescript (a gift from Dr D Evans, Ciba-Geigy, Basle, Switzerland). The PTHrP probe was a 535 bp fragment of PTHrP which had been cloned into pBluescript and sequenced (Walsh *et al.*, 1994). cDNA was labelled with[α-$^{32}$P]dCTP (specific activity 3000 Ci $mmol^{-1}$) using a random primed labelling kit (Boehringer). The blots were prehybridised for 2 h at 42°C in 40% formamide, 5 × SSC, 50 mM phosphate buffer (pH 7), 1 × SDS, 10 × Denhardt's and 200 μg $ml^{-1}$ denatured salmon sperm DNA (Sigma). Denatured probe ($5 \times 10^7$ c.p.m. per membrane) was added directly to the prehybridisation mix and incubated at 42°C overnight. Membranes were washed three times in 0.2 × SSC/1% SDS at 60°C for 20 min and exposed to XO-mat film (Kodak) with an intensifying screen at −70°C.

### *Northern blot analysis*

A 5 μg aliquot of poly(A) + RNA from MCF-7 cells was fractionated on a formaldehyde agarose gel and transferred to Hybond N (Amersham). The membrane was then prehybridised, hybridised and washed as described previously. The membrane was then exposed to XO-mat film (Kodak) with an intensifying screen at −70°C for 5 days.

### *Measurement of thymidine incorporation*

Cells were detached from the culture dish with 0.25% trypsin, resuspended in DMEM/10% FCS and seeded into a 96-well plate at $5 \times 10^4$ cells per well. The plates were incubated at 37°C for 72 h to ensure that all wells were confluent, the medium removed and the wells washed once in serum-free DMEM/0.1% bovine serum albumin (BSA) and 100 μl of fresh serum-free DMEM/0.1% BSA was added to each well. The wells were incubated for a further 4 days in serum-free medium, after which the medium was again replaced with 100 μl of fresh medium/0.1% BSA containing various concentrations of PTH(1-34) or PTHrP(1-34) (both Peninsula Labs). A 0.5 μCi aliquot of [$^3$H]thymidine was then added to each well and the plates were incubated for a further 24 h. The plates were harvested with a cell harvester and [$^3$H]thymidine incorporation measured by scintillation counting.

### *Measurement of bromodeoxyuridine uptake*

Uptake of bromodeoxyuridine (BrdU) by MCF-7 cells was assessed by immunocytochemical staining using an Amersham cell proliferation kit (Amersham, UK). PTHrP (100 ng $ml^{-1}$) was added to serum-free cultures of MCF-7 cells. After 20 h incubation at 37°C bromodeoxyuridine labelling reagent was added. After a further 4 h incubation the medium was removed from the dishes and the cell layers were fixed in 90% ethanol/10% acetic acid. Immunocytochemical detection of BrdU was then carried out using mouse anti-BrdU followed by horseradish peroxidase-coupled rat anti-mouse immunoglobulin and 2,3-diaminobenzidine as substrate. The dishes were examined microscopically and the percentage of dark-staining nuclei recorded in each field. Three wells were used for each treatment and ten fields examined in each well.

### *Measurement of intracellular cyclic AMP levels*

The cell line(s) to be investigated were seeded in DMEM/10% FCS in six-well plates and grown to confluence. The medium was then removed and replaced with serum-free DMEM containing the phosphodiesterase inhibitor 1μM 3-isobutyl-1-methyl-xanthine (IBMX) (Sigma) and PTHrP as appropriate. After 20 min incubation with or without PTHrP, the medium was removed and 0.5 ml of 60% ethanol added to each well. The cell layer was scraped into the ethanol with a plastic transfer pipette and transferred to a 1.5 ml Eppendorf tube. The sample was then acetylated and cAMP level measured by competitive radioimmunoassay as previously described (O'Reilly *et al.*, 1986).

### *Statistical analysis*

Statistical analysis was carried out by analysis of variance (ANOVA), and significance between groups determined by Duncan's new multiple range test.

### Results

Figure 1a shows RT-PCR analysis of PTH/PTHrP receptor using cDNA from MCF-7, ZR-75-1, T-47-D, SK-BR-3, Hs578T, MDA-MB231 and Saos-2 cells as template. The quality of the cDNA had previously been verified by detection of several transcripts, including β-actin and glyceraldehyde-3-phosphate dehydrogenase (data not shown). Bands of the predicted 571 bp were visible in the PCR products from Saos-2, MCF-7 and SK-BR-3 cells. There were no products visible in the reactions using ZR-75-1, T-47-D, Hs578T and MDA-MB231 cDNA as template or in negative controls. The products from Figure 1a were blotted onto a nylon membrane and probed with a $^{32}$P-labelled PTH/PTHrP receptor cDNA (Figure 1b). Specific hybridisation was observed with the 571 bp products from Saos-2, MCF-7 and SK-BR-3, confirming the presence of PTH/PTHrP receptor transcripts. Additionally, weak Southern blot signals were observed with MDA-MB231 and possibly T-47D cDNA. The expression of the PTH/PTHrP receptor by MCF-7 cells was also demonstrated by Northern blot analysis (Figure 2).

The breast cancer cell lines were also investigated for the expression of PTHrP mRNA. RT-PCR identified specific 535 bp products in Saos-2, MCF-7, ZR-75-1, T-47-D and Hs578T cells (Figure 3a), which was confirmed by Southern blot analysis (Figure 3b).

Further studies of MCF-7 and Hs578T were performed in order to determine whether the presence of PTH/PTHrP

**Figure 1** (**a**)Total RNA was isolated from confluent cultures of Saos-2, MCF-7, ZR-75-1, T-47-D, SK-BR-3, Hs578T and MDA-MB231 cells, and used in RT–PCR with primers specific for the PTH/PTHrP receptor (see Materials and methods). Lane 1, 1 kb marker (Gibco-BRL); lane 2, RT–PCR of Saos-2 cDNA showing specific amplification of a 571 bp product; lane 3, MCF-7 PCR products; lane 4, ZR-75-1 PCR products; lane 5, T-47-D PCR products; lane 6, SK-BR-3 PCR products; lane 7, Hs578T PCR products; lane 8, MDA-MB231 PCR products; lane 9, PCR of no DNA negative control. (**b**) Southern blot of **a** probed with a $^{32}$P-labelled PTH/PTHrP receptor cDNA. Lane 1, 1 kb marker (Gibco-BRL); lane 2, RT–PCR of Saos-2 cDNA showing specific amplification of a 571 bp product; lane 3, MCF-7 PCR products; lane 4, ZR-75-1 PCR products; lane 5, T-47-D PCR products; lane 6, SK-BR-3 PCR products; lane 7, Hs578T PCR products; lane 8, MDA-MB231 PCR products; lane 9, PCR of no DNA negative control.

**Figure 2** Northern blot hybridisation of 5 μg of MCF-7 poly(A)$^+$RNA with a $^{32}$P-labelled PTH/PTHrP receptor probe.

**Figure 3** (**a**)Total RNA was isolated from confluent cultures of Saos-2, MCF-7, ZR-75-1, T-47-D, SK-BR-3, Hs578T and MDA-MB231 cells, and used in RT–PCR with primers specific for PTHrP (see Materials and methods). Lane 1, 1 kb marker (Gibco-BRL); lane 2, RT–PCR of Saos-2 cDNA showing specific amplification of a 571 bp product; lane 3, MCF-7 PCR products; lane 4, ZR-75-1 PCR products; lane 5, T-47-D PCR products; lane 6, SK-BR-3 PCR products; lane 7, Hs578T PCR products; lane 8, MDA-MB231 PCR products; lane 9, PCR of no DNA negative control. (**b**) Southern blot of **a** probed with a $^{32}$P-labelled PTHrP cDNA. Lane 1, 1 kb marker (Gibco-BRL); lane 2, RT–PCR of Saos-2 cDNA showing specific amplification of a 57 bp product; lane 3, MCF-7 PCR products; lane 4, ZR-75-1 PCR products; lane 5, T-47-D PCR products; lane 6, SK-BR-3 PCR products; lane 7, Hs578T PCR products; lane 8, MDA-MB231 PCR products; lane 9, PCR of no DNA negative control.

receptor mRNA was accompanied by the expression of a functional receptor. Proliferation studies in response to PTH(1–34) and PTHrP(1–34) were performed on MCF-7 and Hs578T cells by [$^3$H]thymidine incorporation. Neither peptide had any effect when added to rapidly proliferating cells (data not shown). However, when the cells were made quiescent by incubation in serum-free medium at confluence, MCF-7 cells (Figure 4) proliferated in a dose-dependent manner in response to PTHrP(1–34). All doses of PTHrP between 1.6 and 100 ng ml$^{-1}$ significantly stimulated [$^3$H]thymidine uptake. In other experiments where PTH and PTHrP were directly compared, the two molecules were approximately equipotent in the stimulation of proliferation of MCF-7 cells (Figure 5), but neither peptide increased the uptake of [$^3$H]thymidine by Hs578T cells (Figure 6). Additionally, to confirm the [$^3$H]thymidine data, studies were performed on the uptake of BrdU by MCF-7 cells. Treatment of cultures for 20 h with PTHrP (100 ng ml$^{-1}$) resulted in 12.2 ± 3% (mean ± s.e.m.) of nuclei stained compared with 2.6 ± 1% for control cultures.

Changes in intracellular cAMP levels were measured in MCF-7 and Hs578T cells in response to PTHrP(1–34). In MCF-7 cells there was a significant increase in the level of intracellular cAMP when cultures were treated with 100 ng ml$^{-1}$ PTHrP(1–34) but no elevation at 10 ng ml$^{-1}$ (Figure 7). In experiments with Hs578T cells (data not shown) there was no significant change in the concentrations of intracellular cAMP.

### Discussion

This study has demonstrated that transcripts for the PTH/PTHrP receptor can be detected by RT–PCR in the human breast cancer cell lines, MCF-7 and SK-BR-3 and the osteosarcoma cell line, Saos-2. In addition, Southern blot analysis detected receptor expression in MDA-MB231 and possibly in T-47-D cells. The receptor was not identified in ZR-75-1 or Hs578T cells. PTH and PTHrP were reportedly

**Figure 4** Effect of PTHrP on [$^3$H]thymidine incorporation by MCR-7 cells. MCF-7 cells were seeded into a 96-well plate at $5 \times 10^4$ cells per well and grown to confluence. The cells were then incubated in serum-free medium for 4 days. The medium was then replaced with fresh medium containing 0.4–100 ng ml$^{-1}$ PTHrP(1–34) and 0.5 $\mu$Ci of [$^3$H]thymidine. The plates were incubated for a further 24 h, cells harvested and [$^3$H]thymidine incorporation measured by scintillation counting. All data represented as means + s.e.m. ($n = 6$). Asterisk denotes significance ($P < 0.05$).

**Figure 6** Effect of PTH (■) and PTHrP (□) on [$^3$H]thymidine incorporation by Hs578T cells. Hs578T cells were seeded into a 96-well plate at $5 \times 10^4$ cells per well and grown to confluence. The cells were then incubated in serum-free medium for 4 days. The medium was then replaced with fresh medium containing 0.4–100 ng ml$^{-1}$ PTH(1–34) or PTHrP(1–34) and 0.5 $\mu$Ci of [$^3$H]thymidine. The plates were incubated for a further 24 h, cells harvested and [$^3$H]thymidine incorporation measured by scintillation counting. All data represented as means + s.e.m. ($n = 3$).

**Figure 5** Effect of PTH (■) and PTHrP (□) on [$^3$H]thymidine incorporation by MCF-7 cells. MCF-7 cells were seeded into a 96-well plate at $5 \times 10^4$ cells per well and grown to confluence. The cells were then incubated in serum-free medium for 4 days. The medium was then replaced with fresh medium containing 0.4–100 ng ml$^{-1}$ PTH(1–34) or PTHrP(1–34) and 0.5 $\mu$Ci of [$^3$H]thymidine. The plates were incubated for a further 24 h, cells harvested and [$^3$H]thymidine incorporation measured by scintillation counting. All data represented as means + s.e.m. ($n = 3$).

**Figure 7** Effect of PTHrP(1–34) on intracellular cAMP in MCF-7 cells. Cells were grown to confluence in six-well plates and incubated with IBMX/PTHrP for 20 min. Cell layers were harvested as described in Materials and methods and cAMP determined by RIA. All data represented as mean + standard deviation ($n = 6$). Asterisk denotes significance ($P < 0.05$).

detected in cultures of breast cancer cells isolated from a patient with humoral hypercalcaemia of malignancy (Francini *et al.*, 1993), and we have previously detected PTHrP transcripts in MCF-7 and Hs578T cells and immunoreactive peptide in Hs578T cells but not in MCF-7 cells (Walsh *et al.*, 1992). In the data presented here transcripts for PTHrP were detected in MCF-7, ZR-75-1, T-47-D and Hs578T cells. The failure to detect PTH/PTHrP receptor mRNA in ZR-75-1 and Hs578T cells may be due to constitutive lack of expression or, alternatively, down-regulation as a result of PTHrP production, as has been suggested from studies of rat vascular tissue in which high PTHrP mRNA levels are associated with low PTH/PTHrP transcripts and vice versa (Okano *et al.*, 1994). Further study is required to determine if endogenous production of PTHrP can modulate the expression of PTH/PTHrP receptor in breast cancer cells.

PTH(1–34) and PTHrP(1–34) modulated the proliferation of MCF-7 cells but had no effect on Hs578T cells. Furthermore, in response to PTHrP(1–34) (100 ng ml$^{-1}$), intracellular levels of cAMP were elevated in MCF-7 cells but not Hs578T cells. PTH(1–34) and PTHrP(1–34) stimulated [$^3$H]thymidine incorporation by MCF-7 cells equipotently, suggesting that both hormones mediate their effects through the same receptor. PTH and/or PTHrP has previously been reported to stimulate the proliferation of other cell types, including osteoblasts (MacDonald *et al.*, 1986), human renal carcinoma cells (Burton *et al.*, 1990) and Walker 256 carcinoma cells (Benitez-Verguizas and Esbrit, 1994).

These results indicate that PTH and PTHrP may be growth factors for breast cancer cells and may play a significant role in the progression of malignancy. PTH and

PTHrP have been shown to stimulate the production of matrix-degrading enzymes, including plasminogen activator (PA), in other cell types (Hamilton et al., 1984). If PA production was linked to PTH/PTHrP receptor activation in breast cancer cells this would have major implications for metastasis. In addition, other work has shown that PTHrP expression by MDA-231 cells is stimulated by an extracellular matrix produced by bone cells (Guise et al., 1994.

PTH/PTHrP receptor transcripts have an apparent widespread tissue distribution (Urena et al., 1993), and we have shown that breast cancer cells express the receptor and respond to PTH/PTHrP. There is some evidence that primary hyperparathyroidism can be associated with increased incidence of malignancies (Farr et al., 1973). A mitogenic effect of PTH on cancer cells could be one mechanism to explain this observation, but further work is required to confirm this.

Subclones of the 8701 BC primary breast cancer cell line can be subdivided according to their ability to express PTHrP mRNA, with PTHrP-positive clones displaying a more aggressive growth behaviour than PTHrP-negative clones *in vitro* (Luparello et al., 1993). In addition, an RT–PCR study of 38 normocalcaemic breast cancer patients with long-term follow-up demonstrated higher levels of PTHrP expression in patients who subsequently developed bone metastases (Bouizar et al., 1993). In the data presented here we have shown the presence of PTH/PTHrP receptor mRNA in four out of six breast cancer cell lines, and one of these, MCF-7, was shown to proliferate in response to PTH(1–34) and PTHrP(1–34). We conclude that PTHrP may be an important autocrine/paracrine factor in the progression of breast cancer.

**Acknowledgements**
This work has been supported by the North West Cancer Research Fund (UK) The authors wish to thank Dr G Bilbe (Ciba-Geigy, Basle) for the PCR primers and Dr D Evans (Ciba-Geigy, Basle) for the PTH/PTHrP receptor probe. We are also grateful to Mrs B Wlodarski and Ms J Dillon for technical assistance and Mr D Bassi for photography.

**References**

ABOU-SAMRA AB, JUPPNER H, FORCE T, FREEMAN MW, KONG XF, SCHIPANI E, URENA P, RICHARDS J, BONVENTRE JV, POTTS JT, KRONENBERG HM AND SEGRE GV. (1992). Expression cloning of a common receptor for parathyroid hormone and parathyroid hormone-related peptide from rat osteoblast-like cells: a single receptor stimulates intracellular accumulation of both cAMP and inositol triphosphates and increases intracellular free calcium. *Proc. Natl Acad. Sci. USA*, **89**, 2732–2736.

BENITEZ-VERGUIZAS J AND ESBRIT P. (1994). Proliferative effect of parathyroid-related protein on the hypercalcaemic Walker 256 carcinoma cell line. *Biochem. Biophys Res. Commun.*, **198**, 1281–1289.

BOUIZAR Z, SPYRATOS F, DEYTIEUX S, DE VERNEJOUL M AND JULLIENE A. (1993). Polymerase chain reaction analysis of parathyroid hormone-related protein gene expression in breast cancer patients and occurrence in bone metastases. *Cancer Res.*, **53**, 5076–5078.

BROADUS AE, MANGIN M, IKEDA K, INSOGNA KL, WEIR EC, BURTIS WJ AND STEWART AF. (1988). Humoral hypercalcaemia of cancer: identification of a novel parathyroid hormone-like peptide. *N. Engl. J. Med.*, **319**, 556–563.

BUNDRED NJ, RATCLIFFE WA, WALKER RA, COLEY S, MORRISON JM AND RATCLIFFE JG. (1991). Parathyroid hormone related-protein and hypercalcaemia in breast cancer. *Br. Med. J.*, **303**, 1506–1509.

BURTIS WJ, WU J, BUNCH CM, WYSOLMERSKI JJ, INSOGNA KI, WEIR EC, BROADUS AE AND STEWART AF. (1987). Identification of a novel 17,000-dalton parathyroid hormone-like adenylate cyclase-stimulating protein from a tumour associated with humoral hypercalcemia of malignancy. *J. Biol. Chem.*, **262**, 7151–7156.

BURTON PB, MONIZ C AND KNIGHT DE. (1990). Parathyroid hormone related peptide can function as an autocrine growth factor in human renal cell carcinoma. *Biochem. Biophys. Res. Commun.*, **167**, 1134–1138.

CHASE, LR, FEDAK SA AND AURBACH GD. (1969). Activation of skeletal adenyl cyclase by parathyroid hormone in vitro. *Endocrinology*, **84**, 761–768.

CHOMCZYNSKI P AND SACCHI N. (1987). Single-step method of RNA isolation by acid guanidinium thiocyanate–phenol–chloroform extraction. *Anal. Biochem.*, **162**, 156–159.

COSMAN F, MORROW B, KOPAL M AND BILEZIKIAN JP. (1989). Stimulation of inositol phosphate formation by ROS 17/2.8 cell membranes by guanine nucleotide, calcium and parathyroid hormone. *J. Bone. Miner. Res.*, **4**, 413–420.

DONAHUE HJ, FRYER MJ, ERIKSEN EF AND HEATH H. (1988). Differential effects of parathyoid hormone and its analogues on cytosolic calcium ion and cAMP levels in cultured osteoblast-like cells. *J. Biol. Chem.*, **263**, 13522–13527.

EVELY RS, BONOMO A, SCHNEIDER HG, MOSELY JM, GALLAGHER JA AND MARTIN TJ. (1990). Structural requirements for the action of PTHrP on bone resorption by isolated osteoclasts. *J. Bone Miner Res.*, **6**, 85–93.

FARR HW, FAHEY TJ, NASH AG AND FARR CM. (1973). Primary hyperparathyroidism and cancer. *Am. J. Surg.*, **126**, 539–543.

FERRARI SI, RIZZOLI R AND BONJOUR JP. (1992). Parathyroid hormone related protein production by primary cultures of mammary epithelial cells. *J. Cell Physiol.* **150**, 304–311.

FRANCINI G, MAIOLOI E, PETRIOLI E, PAFFETTI P, GONNELLI S AND AQUINO A. (1993). Production of parathyroid hormone and parathyroid hormone-related protein by breast cancer cells in culture. *J. Cancer Res. Clin. Oncol.*, **119**, 421–425.

GOLTZMAN D, HENDY GN AND BANVILLE D. (1989). Parathyroid hormone-like peptide: molecular characterization and biological properties. *Trends Endocrinol. Metab.*, **1**: 39–44.

GUISE TA, TAYLOR SD, YONEDA T, SASAKI A, WRIGHT K, BOYCE BF, CHIRGWIN JM AND MUNDY GR. (1994). Parathyroid hormone-related protein (PTHrP) expression by breast cancer cells enhance osteolytic bone metastases in vivo (abstract). *J. Bone Miner Res.*, **9**, (Suppl. 1), 30.

HAMILTON JA, LINGELBACH SR, PARTRIDGE NC AND MARTIN TJ. (1986). Regulation of plasminogen activator production by bone-resorbing hormones in normal and malignant osteoblasts. *Endocrinology*, **116**, 2186–2191.

HORIUCHI N, CAULFIELD MP, FISHER JE, GOLDMAN ME, McKEE RL, REAGEN JE, LEVY JJ, NUTT RF, RODAN SB, SCHOFIELD TI, CLEMENS TL AND ROSENBLATT M. (1987). Similarity of synthetic peptide from human tumour to parathyroid hormone in vivo and in vitro. *Science*, **238**, 1566–1568.

LUPARELLO C, GINTY AF, GALLAGHER JA, PUCCI-MINAFRA I AND MINAFRA S. (1993). Transforming growth factor $\beta$- 1, 2, and 3, urokinase and parthyroid hormone related peptide expression in 8701-BC breast cancer cells and clones. *Differentiation* **55**, 73–80.

MACDONALD BR, GALLAGHER JA AND RUSSELL RGG. (1986). Parathyroid hormone stimulates the proliferation of cells derived from human bone. *Endocrinology*, **118**, 2445–2449.

MARTIN TJ AND SUVA LJ. (1989). Parathyroid hormone-related protein in hypercalcaemia of malignancy. *Clin. Endocrinol.*, **31**, 631–647.

MONIZ C, BURTON PBJ, MALIK AN, DIXIT M, BANGA JP, NICOLAIDES K, QUIRKE P, KNIGHT DE AND McGREGOR AM. (1990). Parathyroid hormone-related peptide in normal human fetal development. *J. Mol. Endocrinol.*, **5**, 259–266.

MOSELEY JM, KUBOTA M, DIEFENBACH-JAGGER H, WETTENHALL REH, KEMP BE, SUVA LJ, RODDA CP, EBELING PR, HUDSON PJ, ZAJAC JD AND MARTIN TJ. (1987). Parathyroid hormone-related protein purified from a human lung cancer cell line. *Proc. Natl Acad. Sci. USA*, **84**, 5048–5052.

OKANO K, WU S, HUANG X, PIROLA CJ, JUPPNER H, ABOU-SAMRA A, SEGRE GV, IWASAKI K, FAGIN JA AND CLEMENS TL. (1994). Parathyroid hormone (PTH)/PTH-related protein (PTHrP) receptor and its messenger ribonucleic acid in rat aortic vascular smooth muscle cells and UMR osteoblast-like cells: cell-specific regulation by angiotensin-II and PTHrP. *Endocrinology*, **135**, 1093–1099.

O'REILLY DSJ, FRASER WD AND HUTCHINSON AS. (1986). The effect of insulin on the renal responses to parathyroid-hormone in man. *J. Endocrinol.*, **108**, 256.

POWELL GJ, SOUTHBY J, DANKS JA, STILLWELL RG, HAYMAN JA, HENDERSON MA, BENNETT RC AND MARTIN TJ. (1991). Localisation of parathyroid hormone-related protein in breast cancer metastases: Increased incidence in bone compared with other sites. *Cancer Res.*, **51**, 3059–3061.

SCHOEFL C, CUTHBERTSON KSR, GALLAGHER JA, PENNINGTON SR, COBBOLD PH, HESCH RD, BRABANT G AND VON ZUR MUHLEN A. (1991). Measurement of intracellular calcium in single aequorin injected and suspensions of fura-2 loaded ROS 17/2.8 cells and normal human osteoblasts: effect of parathyroid hormone. *Biochem. J.* **274**, 15–20.

SOUTHBY J, KISSIN MW, DANKS JA, HAYMAN JA, MOSELEY JM, HENDERSON MA, BENNETT RC AND MARTIN TJ. (1990). Immunohistochemical localisation of parathyroid hormone-related protein in human breast cancer. *Cancer Res.*, **50**, 7710–7716.

STEWART AF, WU T, GOUMAS D, BURTIS WJ AND BROADUS AE. (1987). N-terminal amino acid sequence of two novel tumor-derived adenylate cyclase-stimulating proteins: identification of parathyroid hormone-like and parathyroid hormone-unlike domains. *Biochem. Biophys. Res. Commun.*, **146**, 672–678.

STREWLER GJ, STERN PH, JACOBS JW, EVELOFF J, KLEIN RF, LEUNG SC, ROSENBLATT M AND NISSENSON RA. (1987). Parathyroid hormone-like protein from human renal carcinoma cells: structural and functional homology with parathyroid hormone. *J. Clin. Invest.*, **80**, 1803–1807.

THIEDE MA AND RODAN GA. (1988). Expression of a calcium-mobilising parathyroid hormone-like peptide in lactating mammary tissues. *Science*, **242**, 278–280.

URENA P, KONG XF, ABOU-SAMRA AB, JUPPNER H, KRONENBERG HM, POTTS JT AND SEGRE GV. (1993). Parathyroid hormone (PTH)/PTH-related peptide receptor messenger ribonucleic acids are widely distributed in rat tissues. *Endocrinology*, **133**, 617–623.

VARGAS SJ, GILLESPIE MT, POWELL GJ, SOUTHBY J, DANKS JA, MOSELEY JM AND MARTIN TJ. (1992). Localisation of parathyroid hormone-related protein mRNA expression in breast cancer and metastatic lesions by in situ hybridisation. *J. Bone Miner Res.*, **7**, 971–979.

WALSH CA, GALLAGHER JA, LUPARELLO C, PUCCI-MINAFRA I AND MINAFRA S. (1992). PTHrP production by human breast cancer cell lines in vitro (abstract). *Bone*, **13**, 107.

WALSH CA, BIRCH MA, FRASER WD, ROBINSON J, LAWTON R, DORGAN J, KLENERMAN L AND GALLAGHER JA. (1994). Primary cultures of human osteoblasts produce parathyroid hormone-related protein. *Bone Mineral*, **27**, 43–50.

WYSOLMERSKI J, DAIFOTIS A, BROADUS A AND MILSTONE L. (1993). Overexpression of PTHrP in transgenic mice results in breast hypoplasia (abstract). *J. Bone Miner Res.*, **8**, (Suppl.), 129.